# Enhancing Clinical Decision-Making in Complex Corneal Disorders: The Role of In-Vivo Confocal Microscopy

**DOI:** 10.3390/life13030679

**Published:** 2023-03-02

**Authors:** Alberto Recchioni, Ankur Barua, Alberto Dominguez-Vicent

**Affiliations:** 1 Academic Unit of Ophthalmology, Institute of Inflammation and Ageing, University of Birmingham, Birmingham B15 2SQ, UK; 2 Optometry & Vision Sciences Group, School of Life & Health Sciences, Aston University, Birmingham B4 7ET, UK; 3 Department of Ophthalmology, Birmingham and Midland Eye Centre, Birmingham B18 7QH, UK; 4 Division of Eye and Vision, Department of Clinical Neuroscience, Karolinska Institute, 171 77 Stockholm, Sweden

**Keywords:** in-vivo confocal microscopy, anterior eye, microbial keratitis, acanthamoeba keratitis, fungal keratitis, neuropathic pain, dry eye

## Abstract

This study aims to describe how in-vivo confocal microscopy (ICVM) results improved diagnosis and treatment in three patients with complex corneal disorders at a single institution. Case one was a 36-year-old woman contact lens wearer referred to the hospital eye service (HES) by her community optician for a suspected corneal ulcer in her left eye. The case demonstrated that where laboratory cell culture was inconclusive, IVCM imaging improved diagnosis and more importantly adjusted the initial treatment till the complete resolution of the case. Case two was a shared-care 66-year-old keratoconus patient under a complex immunosuppression regime who had developed a recent series of post-surgical complications of fungal origin and was experiencing eye pain. IVCM was able to differentiate between an immune-mediated response and fungal keratitis and guide the clinicians towards an optimized treatment. Case three was a long-standing dry eye disease in a 64-year-old woman diagnosed with primary Sjögren’s syndrome where previous treatments failed to improve her symptomatology. IVCM was crucial for prescribing allogeneic serum eyedrops by anticipating early immune changes in the sub-basal corneal nerve plexus. In-vivo confocal microscopy can be an essential non-invasive imaging technique for improving clinicians’ diagnostic precision by adding a layer of certainty that other techniques may lack. Additionally, IVCM allows adjustment of the treatment accordingly, by instantly following any pathologic changes at the cellular level.

## 1. Introduction

During the last decades, many advances in ophthalmic imaging emerged, allowing a reliable, non-contact, in-vivo imaging of ocular structures [1]. The availability of high-resolution imaging of the cornea with different techniques provides new possibilities and additional information that could help in the early diagnosis, and evaluation of progression of different corneal diseases. It is also known that identifying a corneal pathology at its earliest stages is important for preventing more noticeable deterioration of visual acuity and optimizing the long-term prognosis. Concretely, microscopic en-face images of the living human corneal layers have revolutionized the observation, diagnosis and treatment of numerous eye conditions [2,3]. In the UK in 2013, over 2 million people were living with sight loss that accounted for £28.1 million spent from the budget of the national health system (NHS) per year [4]. Among the causes of sight loss, complex corneal disorders such as infections and neuropathies are evident with the former reported with 2617 admissions in UK public hospitals in 2005 [5]. Microbial keratitis (MK) is an eye infection that affects the anterior eye, can potentially lead to sight loss due to corneal opacification and it has been noted to be one of commonest reasons for visiting an emergency department in the UK. In this context, contact lens (CL) wear can play a crucial role as a risk for developing MK, especially on those patients who are wearing CL in extended modality (continuously for up to six nights and seven days) [6]. Some of the most common CL-MK-related causes are: showering with CLs, sleeping with CLs, lack of hand hygiene, lack of follow-up visits, etc. [7].

The Birmingham and Midland Eye Centre (BMEC) is one of the largest UK facilities that receives referrals from other hospitals, general practitioners (GPs) and eye care practices across the West Midlands and nationally. Additionally, it offers seven-days-a-week Eye Casualty services for adults and children with acute sight threatening eye disease. Previous data obtained from 2015–2016 [8] reported that BMEC recorded approximately 120 attendances per day with up to 112.5 admissions per year due to MK with direct costs of admission of £3681 per patient. Consequently, it is evident that complex corneal disorders can negatively impact on the NHS, economy and also on patients’ quality of life [9]. More importantly, these conditions are challenging threats for both clinicians and researchers as they can be potentially misdiagnosed or mistreated [10].

Since its introduction in 1990, in-vivo confocal microscopy (IVCM) has evolved through several advanced technologies to detail the anterior eye ranging from the tandem-scanning confocal microscope up the most recent white light slit-scanning confocal microscope and laser-scanning confocal microscope. The latter, by the means of a laser scanning source at 670 nm in the red wavelength spectrum, is able to acquire images of 400 µm × 400 µm with a resolution in the order of 2–4 µm (e.g., central corneal basal epithelial cells size is approximately 17 μm). Nevertheless, laser scanning IVCM is a minimally invasive imaging technique that has been shown to provide good sensitivity and specificity (55.8% and 84.2%, respectively) in several types of MK [11]. Nowadays, most of the hospital premises that are receiving suspected MK referrals or patients attending their A and E services are basing their diagnosis on culture (positive and negative) together with polymerase chain reaction (PCR) or agar plates. This is despite, these microbiological sample analyses showing poor sensitivity [12], requiring time (sometimes days) to be processed, or being limited in cases of slow-growing fungi and Acanthamoeba. Time for culture diagnosis, access to culture medium and microbiology labs and prior use of anti-infective agents may limit acute management of what can be very aggressive disease processes. The sooner the initial empirical treatment is consolidated to target the pathology, the more likely there will be a better outcome. Ting et al. [13] summarized 10-years of results in fungal keratitis (FK) patients in the UK: 81.2% of 117 subjects included required hospitalization for intensive treatment with a mean hospitalization of 18.9 ± 16.3 days. Additionally, nearly 25% of the total cohort reported a final corrected distance visual acuity of perception of light or worse with up to 9.4% of cases where eye enucleation was required. In light of these results, again IVCM can play a role in disclosing the presence (or not) of FK by supporting clinicians to achieve better post-infection outcomes.

During the last years, IVCM has become widely popular in ocular surface disease (OSD) and dry eye disease (DED). In a recent review by Sim et al. [14], the authors reported that despite potential limitations, such as limited field of view, lack of standardized protocol for image acquisition and quantification, an initial steep learning-curve, IVCM is still considered a supporting tool in diagnosing and managing anterior disorders related to dry eye. More specifically, in DED it appears that the chronic lack of tear film homeostasis might affect the corneal nerve endings by damaging the physiological neurosensory responses [15]. Therefore, it is clear that IVCM can play a role because of its ability to depict the sub-basal corneal nerves plexus (e.g., reduction and abnormalities) and other ocular surface structures (e.g., meibomian glands) [16].

Clinical acumen in the face of complex cases with multi-pathology often leads to empirical treatment and IVCM can be an excellent clinical differentiator in both the acute and chronic settings because it may reveal multiple pathology or deeper pathology which microbiological analysis would not identify. This can be due to the depth of pathology or surface sterilization from prior treatment. These considerations lead to evidence-based treatment directed by findings guiding earlier and more effective treatments.

In this article, we will highlight three ambiguous clinical case examples which have relied on the results from IVCM to direct the clinical course and alter/refine management when alternative diagnostic tools were either indecisive or not available. In all cases, the instrument used was the Heidelberg Retinal Tomograph with a Rostock Corneal Module (HRT-RCM) (Heidelberg Engineering GmbH, Dossenheim, Germany), and the size of all images listed in this work is 400 × 400 microns. All the IVCM scans were performed by the same trained examiner (AR) after the application of a single drop of topical anesthetic (Minims Oxybuprocaine Hydrochloride 0.4%, Bausch & Lomb, Tampa, FL, USA) to reduce the blink reflex and increase patient comfort during the acquisitions. A real-time camera linked to the device was used by the examiner to manually optimize alignment between the cornea and the confocal probe. During the analysis, those images that presented any form of artifact (e.g., excessive compression of the layers/nerves, lack of focus or decentration) were discarded.

## 2. Case Reports

### 2.1. Case 1

**Figure 1:**
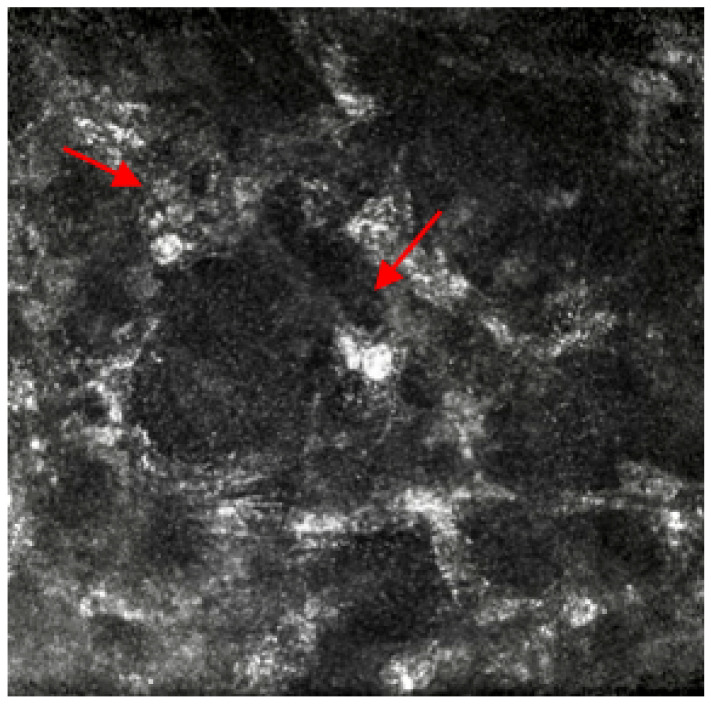
In-vivo confocal microscopy image showing the presence of round double wall cysts and oval-shaped hyper-reflective structures representing Acanthamoeba cysts. Red arrows point at the round double wall cysts.

A contact lens (CL) wearing 36-year-old patient attended a walk-in visit at their local community optician. During this first consultation, the optometrist who saw her reported a red, sore, photosensitive left eye (LE) with the presence of a flat grey infiltrative spot at 5 o’clock, right eye vision 6/6 and LE 6/6^−3^ Snellen scale, and hazy cornea with multiple smaller infiltrative corneal spots. The initial practice management was to advise against CL wear until further notice, plus consider using lubricant eyedrops and lubricant night gel three to four times daily and review in 72 h. At the follow-up visit, while symptoms improved slightly, the same signs were observed, and a Hospital Eye Service (HES) referral was made to rule out the risk of ulcer in LE. HES initial consultation reported worsening of symptoms, vision LE dropped to 6/36 Snellen scale, presence of keratic precipitates and three round infiltrates noted at 4, 5 and 7 o’clock. Immediately, a probable diagnosis of Acanthamoeba keratitis (AK) was made, but a decision to cover for bacterial keratitis was also taken. HES ocular medication prescribed on LE were: g.Brolene four times/day, g.PHMB 0.02% once hourly/day, Chloramphenicol ointment once/day, g. Levofloxacin preservative-free six times/day., PCR culture was also ordered, and a referral was made for IVCM to confirm the current treatment. After the initial week of LE treatment, ocular medication was amended due to the presence of scattered round double wall cysts and oval-shaped hyper-reflective structures representing Acanthamoeba cysts shown by IVCM imaging (Figure 1): g.Brolene, g. polyhexamethylene biguanid 0.02%, g.Levofloxacin six times/day and Chloramphenicol ointment once/day. Unfortunately, PCR reported inconclusive findings. Fortunately, ICVM allowed confirmation of clinical suspicion of AK and it also enabled monitoring of response to AK treatment in the absence of any microbiological culture result. ICVM also confirmed the end of the treatment on the patient’s LE, when vision fully recovered without any further signs and symptoms.

### 2.2. Case 2

**Figure 2:**
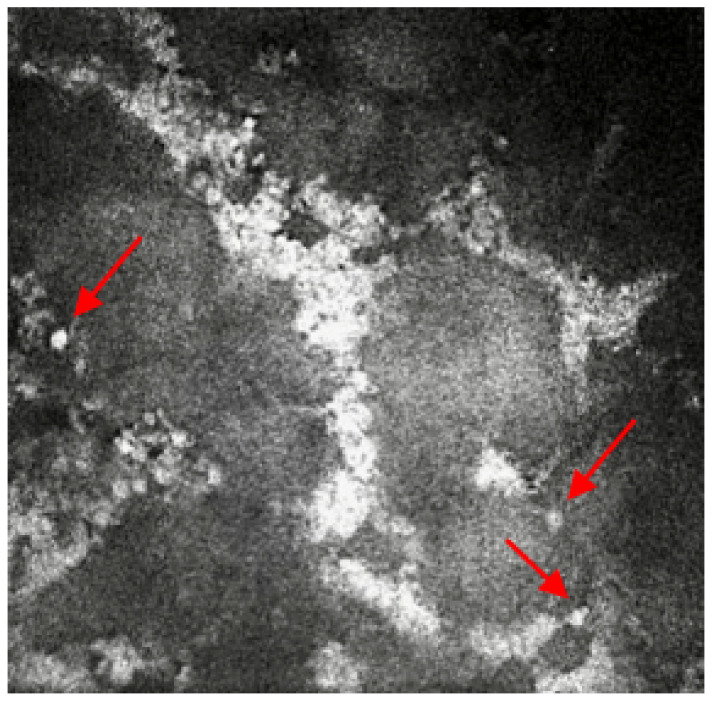
In-vivo confocal microscopy image showing the presence of multiple round bodies, which is highly suggestive of yeast and candida infection. Red arrows point at the round bodies, which are suggestive of yeast and candida infection.

A 66-year-old keratoconic patient with a background of previous corneal grafts to both eyes developed a fungal infection to the right eye after a redo corneal graft 2 years prior. He has chronic plaque psoriasis on methotrexate under dermatology review. After intensive treatment for the fungal infection over 9 months, the decision was taken to offer a redo graft with stem cell transplant. This required modified Cincinnati immunosuppression protocol (mycophenolate, tacrolimus, prednisolone loading dose, acyclovir and cotrimoxazole) [7] but unfortunately, the fungal infection returned following this surgery, He required further excision of the infected tissue with intrastromal and intracameral antifungals. Ocular medication included amphotericin 0.15%, 2 hourly and levofloxacin six times per day. An urgent referral was made for IVCM to help determine whether there was still fungal infection present or if there was an inflammatory reaction against the graft tissue causing the opacification of the cornea with early pthisis and pain. The patient was also on oral immunosuppression and was developing side effects to the medications, so identification of underlying pathology was important. Previous excision of tissue did not reveal any fungal elements and corneal scrapes did not isolate any fungal elements. However, IVCM imaging confirmed the presence of multiple round bodies highly suggestive of yeast and candida infection (Figure 2). This enabled removal of immunosuppressive treatment apart from psoriasis management and a focus on antifungal treatment to avoid spread to surrounding structures despite loss of vision in the affected eye. After 4 months the pain resolved, and the fungal infection settled with no further recurrences. The fungal infection isolated from the initial specimen is extremely rare (Neocucurbitaria unguis-hominis) and IVCM helped confirm presence and allow directed treatment to avoid potential evisceration/enucleation.

### 2.3. Case 3

A 64-year-old patient presented with primary Sjögren’s syndrome (pSS), ocular surface disease with severe dry eyes and conjunctival chalasis. Systemically there was borderline type 2 diabetes (diet controlled), episodes of migraines, neck and shoulder pain attributed to arthritis. Ophthalmologically, the best corrected visual acuity was 0.1 logMAR scale in both eyes.

**Figure 3:**
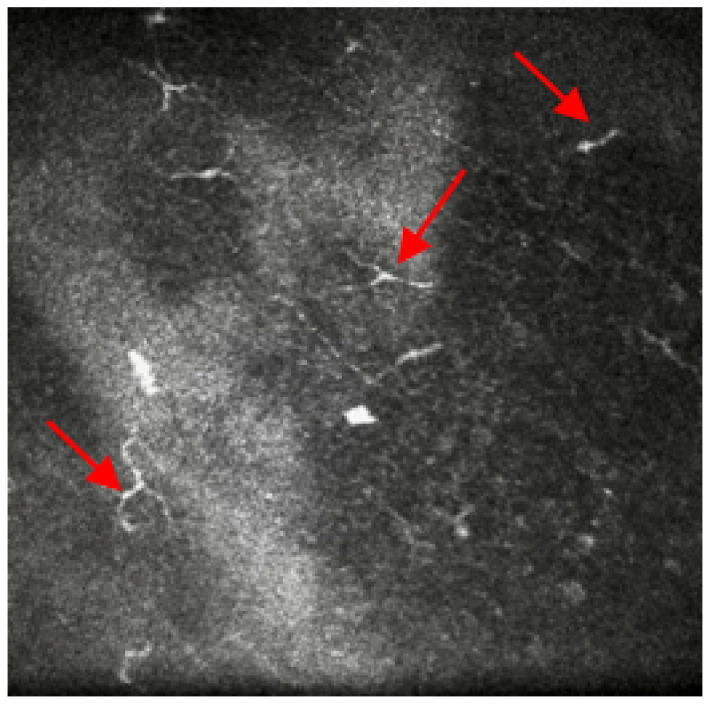
In-vivo confocal microscopy image showing the presence of micro-neuromas at nerve endings. Red arrows point at the micro-neuromas.

Symptoms assessed with the Ocular Surface Disease Index (OSDI) questionnaire reported a score of 41 (moderate), although she referred to constant pain, photophobia (sensitivity to light) and foreign body sensation. Ocular medications prescribed on both eyes were: ocular lubricant (trehalose (3%) and hyaluronic acid) hourly, hyaluronic gel ointment once/night time, cyclosporine eye ointment three times/day. Previously, she has been prescribed preservative-free topical steroids together with Latanoprost for shorter periods (<2 weeks). Insertion of punctum plugs was considered but they frequently fell out with short improvement of her symptoms. Therefore, a referral was made for IVCM to confirm the need for a serum eye drops prescription due to the presence of micro-neuromas at nerve endings (Figure 3).

## 3. Discussion

Currently, the management of complex corneal disorders with multi-pathology requires clinicians to have an array of tools to avoid empirical treatment that often leads to poor outcomes. In the presented article, three different complex clinical cases have been reviewed by pointing out the role of IVCM in corneal analysis. Its clinical importance and relevance in acute and chronic corneal disease cannot be emphasized enough.

Case 1 is a typical case of a CL wearer infective AK that is generally associated with inappropriate lens maintenance and exposure to tap or contaminated water sources [17]. AK is a sight-threatening condition that can lead to blindness with slow progression and can be often misdiagnosed as herpes simplex virus (HSV) or other corneal infections. In case 1, IVCM was able not only to spot and assist inconclusive PCR results but also to guide the clinicians when the treatment had to be terminated, saving the patient from unneeded exposure to the antiamoebic medications and any additional clinical examinations. IVCM is an invaluable tool in AK as it allows supportive and sole diagnosis, check for response to treatment, check for possible cessation of treatment, and analyze possible recurrence of AK if symptoms return.

Case 2 reports on a complex multi-disciplinary case shared between ophthalmology and dermatology: a keratoconus patient with recurrent fungal infections and under immunosuppression treatment for skin disease (psoriasis). To improve the right eye treatment after a recent corneal graft, patient immunosuppression therapy was switched from methotrexate to mycophenolate and tacrolimus (Cincinnati modified protocol). In fact, it is not uncommon to discover unexplained fungal infections linked to an immunosuppression regime [18]. As previously mentioned in another case report by Hassan et al. [19], 31 days is the average between presentation and diagnosis confirmation in UK. Therefore, in this case, fungal disease was co-existent with other infections. The other unusual aspect of this case was the initial fungal infection, which was not found in the excised corneal tissue. This gave false reassurance that the fungus was no longer present. However, IVCM confirmed residual fungal infection allowing cessation of immunosuppressants and directed antifungal treatment.

Case 3 underlines the importance of IVCM as a non-invasive technology to aid in-vivo assessment of structural changes in severe ocular surface disease. During the last decades, relevant research worldwide has considered IVCM to assess morphological changes in the anterior eye related to DED [20]. In particular, the role of corneal nerves in DED patients showed a higher deficit in terms of sub-basal nerve density and their thicknesses. Additionally, as the case presented, it is interesting to observe abnormal findings such as the presence of micro-neuromas at the nerves’ endings that are responsible for the intense stabbing sensation called the *Tinel sign*, also reported in Case 3 ocular symptoms [21]. Previously, Moein, et al. [22] suggested that the presence of corneal neuromas assessed via IVCM might be anticipatory biomarkers for neuropathic corneal pain. Furthermore, IVCM has the ability to spot changes in dendritic cells density and morphology: these increases have shown to be highly correlated with mild DED stages and their image tracking might anticipate early immune changes in more severe stages [23].

In conclusion, IVCM imaging plays a role in providing fine details of the anterior eye, especially in the cornea and related structures. Diagnosis and management of complex eye disorders performed in a timely manner is essential. IVCM’s high-resolution technology can instantly provide this information that otherwise might require additional time through laboratory facilities or might end with inconclusive results (e.g., corneal scraping and cell culture). In this context, it would be helpful for clinicians to further consider IVCM imaging for improving their diagnostic precision in the presence of complex cases that often lead to empirical treatment. Additionally, research centers and academia involved in translational ophthalmology should take IVCM into account in their randomized clinical trials. This would lead to improvements in understanding and also implementing national and international clinical guidelines. Finally, in the near future artificial intelligence-assisted systems will ease the interpretation of IVCM findings by helping clinicians and researchers to tackle its initial steep learning curve.

## Data Availability

Due to the nature of this research, participants of this study did not agree to their data being shared publicly, so supporting data are not available.
